# Editorial: HLA-G in health and disease: comprehensive insights and future therapeutic directions

**DOI:** 10.3389/fimmu.2026.1786277

**Published:** 2026-02-13

**Authors:** Jules Russick, Roberta Rizzo, Ashwin Ajith

**Affiliations:** 1 Comissariat à l'Energie Atomique et aux energies alternatives (CEA), Direction de la Recherche Fondamentale (DRF)-Institut de Biologie Francois Jacob, Service de Recherches en Hémato-Immunologie, Hôpital Saint-Louis, Paris, France; 2 INSERM UMR1342 Institut de Recherche Saint Louis, Université Paris Cité, Paris, France; 3 Department of Environmental and Prevention Sciences, University of Ferrara, Ferrara, Italy; 4 Georgia Cancer Center, Augusta University, Augusta, GA, United States

**Keywords:** HLA-G, immune tolerance, oncology, pregnancy, transplantation

Human leukocyte antigen-G (HLA-G) has emerged as one of the most compelling regulators of immune tolerance. Initially characterized for its essential role at the maternal–fetal interface, HLA-G is now recognized as a key immunomodulatory molecule operating across a wide spectrum of physiological and pathological contexts, including autoimmunity, cancer, transplantation, and chronic inflammation. Over the last decade, HLA-G has shifted from a passive marker to an active regulator of immune responses whose effects depend on genetic background, molecular form, cellular source, and tissue environment. The contributions assembled in this Research Topic capture this evolution in understanding and illustrating how HLA-G biology spans fundamental immunology, mechanistic disease modeling, and clinical application. Together, they trace a coherent translational continuum, from conceptual frameworks, through mechanistic and genetic validation, to clinical relevance, reflecting both the expanding clinical use of HLA-G–related tools and the persistent gaps in our understanding of how this pathway is regulated and functions *in vivo*.

At the conceptual level, this Research Topic emphasizes that HLA-G should no longer be viewed as a singular tolerogenic molecule but rather as a dynamic system integrating multiple layers of regulation. The mini review article by Beli et al. included in this Research Topic provides a comprehensive overview of how HLA-G finely tunes immune responses through its diverse isoforms, alternative splicing patterns, soluble and membrane-bound forms, and interactions with inhibitory receptors. It highlights the duality of HLA-G biology: HLA-G–mediated immune suppression is essential in settings such as pregnancy or transplantation, where controlled tolerance prevents tissue damage, yet the same mechanisms can be co-opted in cancer and infection to promote immune escape and disease progression. By framing HLA-G as a context-dependent checkpoint whose function can be either beneficial or detrimental depending on the pathological setting, this review provides the foundation upon which the subsequent experimental and clinical studies build.

Building on this conceptual foundation, two original studies examined HLA-G as a functional mediator and a putative target in specific pathological contexts. In multiple myeloma, Soncini et al. showed that bone marrow–derived extracellular vesicles are more abundant in patients than in healthy donors and are enriched in HLA-G together with PD-1 and PD-L1, with phenotypic evidence suggesting that these vesicles originate in part from malignant plasma cells and bone marrow immune cells. These vesicles extend the immunosuppressive influence of tumor cells beyond direct cell–cell contact, suppressing T-cell activation and altering cytokine production. Importantly, partial reversal of these effects through HLA-G blockade provides functional evidence that vesicle-associated HLA-G is an active contributor to immune evasion. In the context of kidney transplantation, Hölzenbein et al. focused on the ligand–receptor pair formed by HLA-G and ILT2 (LILRB1) to understand how their genetic variation shapes rejection risk. By analyzing donor HLA-G 3′UTR variants alongside recipient LILRB1 promoter polymorphisms, the authors identify specific haplotype combinations that differentially influence T cell–mediated and antibody-mediated rejections. The differential association of these genetic profiles with T cell–mediated versus antibody-mediated rejection underscores a finely tuned immunogenetic interaction and the importance of considering both ligand availability and receptor expression, thereby providing a framework for more precise risk stratification and personalized immunosuppressive strategies. These results underscore the translational potential of HLA-G/LILRB1 genetic profiling to refine donor selection in living kidney transplantation and to guide the design of next-generation rejection therapies.

The translational dimension of HLA-G biology is amplified when complemented by large-cohort investigations that anchor HLA-G biology in clinically meaningful endpoints. As in transplantation, Miglianti et al. highlighted how subtle changes in the regulatory regions of HLA-G can recalibrate tolerogenic capacity in an organ-specific disease. In primary biliary cholangitis, the analysis of more than 150 patients reveals that specific HLA-G haplotypes, haplotypes especially HLA-G*01:01:01:08/UTR-1, in combination with defined 3′UTR variants, are overrepresented in patients relative to controls and associated with reduced levels of soluble HLA-G and poorer response to standard therapy. These findings imply that inherited variation in the HLA-G locus may contribute to persistent autoimmune liver inflammation and reduced treatment efficacy, and they position HLA-G haplotypes and circulating levels as promising biomarkers for disease prognosis and therapeutic stratification. In colorectal cancer, the demonstration by Zhang et al. that expression of the HLA-G2/6 isoforms in tumor tissue from more than 300 patients, rather than total HLA-G expression, is independently associated with poorer overall survival challenges long-standing assumptions about HLA-G as a uniform entity. This work underscores the critical importance of isoform-specific analysis and reveals that distinct HLA-G variants may exert non-redundant biological effects with direct prognostic relevance. Such findings argue strongly for the integration of refined molecular profiling into future clinical studies and therapeutic decision-making.

This Research Topic provides a rare, nearly continuous picture of how a single immune-regulatory axis can be followed from conceptual therapeutic frameworks to clinically measurable impact. Beginning with conceptual models of immune regulation, progressing through mechanistic and genetic dissection, and culminating in large-scale clinical observations, the studies presented in this Topic collectively demonstrate how an immunoregulatory pathway can influence diverse disease processes, underscore the feasibility and added value of integrating HLA-G–related parameters into clinical practice and position HLA-G as a key element linking immune tolerance, disease susceptibility, and therapeutic response. Importantly, the work assembled here also underscores that this interest in HLA-G, an already clinically relevant molecule, is growing rapidly, driven by advances in immunotherapy, genetics, and biomarker research, while our understanding of its detailed modes of action, isoform-specific signaling, receptor cross-talk, regulation by non-coding regions, and expression on extracellular vesicles, remains incomplete. Closing this gap between extensive clinical exploitation and only partial mechanistic understanding will be crucial to safely and effectively harness HLA-G. This challenge will require translating current advances into rational, context-aware applications that balance the tolerogenic potential of HLA-G against the risk of immune escape, thus supporting its emergence as a precision tool in immune modulation. We anticipate that this Research Topic will stimulate further multidisciplinary research, accelerating the safe and effective integration of HLA-G–centered insights into clinical immunology.

